# Methodological comparison between salivary and plasma inflammatory biomarkers in third molar surgery patients

**DOI:** 10.1186/s12903-025-07368-2

**Published:** 2025-11-22

**Authors:** Lars B. Eriksson, Anders Larsson, Mats Eriksson, Åke Tegelberg, Andreas Thor, Torsten Gordh

**Affiliations:** 1 Department of Surgical Sciences, Uppsala University, Uppsala, Sweden; 2 Centre for Clinical Research, Uppsala University, Falun, Sweden; 3 Department of Medical Science, Uppsala University, Uppsala, Sweden; 4 NOVA Medical School, New University of Lisbon, Lisbon, 1099-085 Portugal; 5 Department of Orofacial pain and jaw function, Faculty of Odontology, Malmö University, Malmö, Sweden; 6 Plastic & Oral and Maxillofacial Surgery, Uppsala University, Uppsala, Sweden; 7 Multidisciplinary Pain Centre, Uppsala University, Uppsala, Sweden; 8 Department of Oral and Maxillofacial Surgery, County Hospital, Falu Lasarett, Falun, 79182 Sweden

**Keywords:** Saliva, Plasma, Biomarkers, Cytokines, Inflammation, Inflammation mediators, Pain

## Abstract

**Background:**

Changes in saliva biomarkers levels may be an inflammatory response to local surgical trauma or other threats. The aim of the study was to compare saliva and plasma regarding the expression of a large set of inflammatory biomarkers to find clinically useful biomarkers in saliva.

**Methods:**

Both saliva and blood samples were collected from 165 individuals. For every patient, the samples were collected on the same occasion. Saliva and plasma protein levels were analysed using the OLINK Proseek inflammation panel measuring 92 cytokines, chemokines, and growth factors (CCGF). The levels of individual CCGF were compared between saliva and plasma. A Spearman rank test was used to find correlations (r_s_).

**Results:**

Of the 92 inflammatory biomarkers, 71 were detected in plasma, 63 in saliva, and 58 in both saliva and plasma. IL-6 (r_s_ = 0.1535, *p* = 0.048) and CST5 (r_s_ = -0.2542, *p* = 0.00098) showed significant correlations between their expression levels in saliva and plasma. Furthermore, 36 significantly correlated heterogeneous cytokine pairs were identified. In only one pair was r_s_ ≥ ± 0.25; in all other cases the correlations were even weaker. CCGF, including IL-8, VEGFA, CDCP1, IL-6, IL-1 alpha, OSM, TNFSF14, CCL28, EN-RAGE, and CASP-8, were expressed much more strongly in saliva than in plasma.

**Conclusion:**

We found major differences in the levels of inflammatory biomarkers in saliva versus plasma when analysed with the OLINK method. A compound of the six most prominent proteins in saliva (IL-8, IL-1 alpha, IL-6, OSM, CST5 and CCL28) are expressed more strikingly in saliva than in plasma. They are also connected by certain inflammatory functions. Obviously, saliva samples do not give the same information on inflammation processes as those found in plasma. This information may be important for future inflammation studies.

**Trial registration:**

EudraCT under number, 2014-004235-39 (29/09/2014). ClinicalTrials.gov, ID: NCT04459377 (15/07/2020).

**Supplementary Information:**

The online version contains supplementary material available at 10.1186/s12903-025-07368-2.

## Background

There are several studies measuring CCFG in peripheral fluids such as plasma, saliva, or urine, but only a few of them have directly compared proteins in saliva versus plasma from the same individuals and at the same time [1–5]. Saliva is easily accessible for sampling with a non-invasive method, in contrast to sampling of blood, cerebrospinal fluid, or even urine, especially when integrity is a concern. CCGF are of the utmost importance in the inflammation process and, thus, crucial for understanding inflammatory pain. The pathogenesis of acute and chronic pain is not fully understood. Through a better understanding of the inflammatory proteins and their associations across different compartments, it may be possible to gain more insight into the complex processes behind nociception and pain experience. The immune system partially acts as a diffuse sense organ, when activated, by signalling the brain about peripheral events [6]. Inflammation is a biological response of the immune system to harmful stimuli such as pathogens, damaged cells, or irritants [7]. In pathological manifestations in the oral cavity - such as in oral potentially malignant disorders including leucoplakia - salivary cytokines are increased; this is particularly evident in oral cancer cases [8, 9]. In one study from 2022, 10 cytokines in saliva vs. plasma were compared in 71 elderly participants, concluding that inflammatory biomarkers in saliva are associated with those found in plasma, which suggests that there is a similar inflammatory mechanism between saliva and plasma [3]. Third molar surgery is a well-established pain model frequently used in pain research especially in pharmacological trials [10–14]; normal findings in saliva and plasma in that population are useful to evaluate the model. To clarify if specific biomarkers quantified in saliva can substitute such quantification in plasma, further scientific comparisons are needed. To our knowledge it has not been made previously in a cohort like ours.

## Patients and methods

The aim of this study was to compare salivary with plasma expression of inflammatory biomarkers using paired samples taken from patients, and to find correlations or proteins primarily expressed at high levels in saliva.

Patients referred to the Department of Oral and Maxillofacial Surgery at the Falu County Hospital, Sweden, for mandibular third molar removal, were considered for inclusion. The study focused on men and women from 18 to 44 years old, with a bodyweight of 50 to 120 kg. Patients who met the exclusion criteria (Table 1.) were not invited to participate in the study. Those who signed a written informed-consent form were then included in the study. All participants fasted from 12.00 pm on the night before the samples of saliva and plasma were collected. These patients took part in a clinical trial, reported elsewhere, but all sampling of data presented in this study was made before the patients were exposed to any intervention [15, 16]. The patients and surgical procedures have been described in detail elsewhere [15].

**Table 1:** Exclusion criteria

Exclusion criteria
Medication: analgesics, hypnotics (the week before surgery), thyroid hormones, psychotropic drugs or MAO-inhibitors.
Hypertension [> 150/95 mmHg at screening], Congestive heart failure, Psychosis, Epilepsy, Hyperthyroidism, Myasthenia gravis, Glaucoma, Verified sleep apnoea, Diabetes (insulin dependent), Porphyria, Pregnancy, Breast-feeding, Blood infections (Hepatitis B, C and HIV), Inability to comprehend written or spoken information, known hypersensitivity or allergy to midazolam, ketamine, ibuprofen or local anaesthetic.

### Saliva collection

**Fig. 1:**
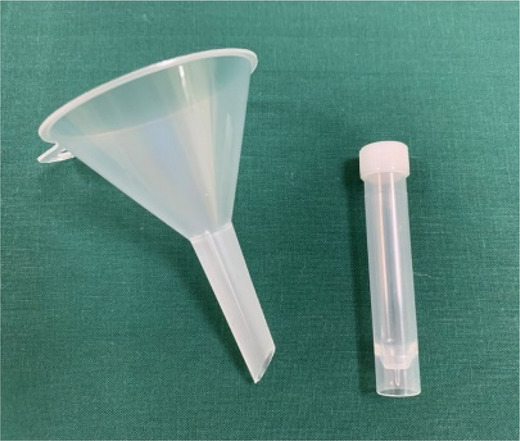
Plastic funnel and Sarstedt^®^ 3 ml tube for saliva sampling

The saliva samples were collected between 8 am and 2 pm by asking each patient to spit into a funnel, fitted in a plastic tube (Sarstedt^®^ 3 ml), to reach at least 1 ml saliva (Fig. 1). Within one hour, the samples were frozen and stored at −80 °C until they were analysed.

### Plasma collection

**Fig. 2:**
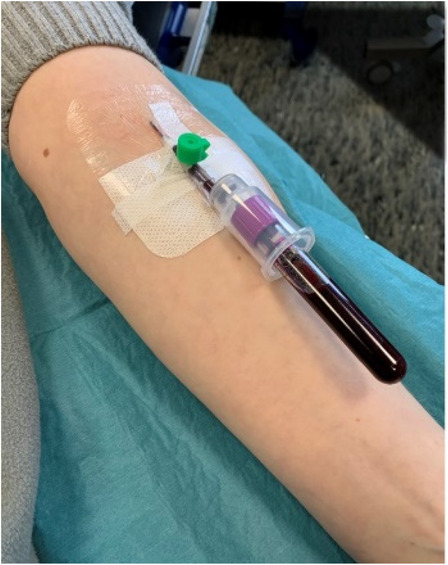
Sampling of venous blood from superficial arm vein

Plasma samples were collected immediately after the saliva collection by placing an intravenous (IV) cannula (BD Venflon^®^ Ø 1.3 mm) into a superficial vein on the back of the hand or on the forearm (Fig. 2). The blood was collected in EDTA tubes (BD Vacutainer^®^). The samples were prepared by centrifugation for 5 min at 2400 g, and the plasma portions were stored in aliquots at −80 °C within one hour from sampling until the analysis took place. Preparation time affects a majority of proteins measured in plasma and there is a risk of causing pre-analytic errors when the centrifugation is delayed, especially by more than 1 h [17, 18]. 165 patients were enrolled, resulting in 165 paired samples (*n* = 330) of plasma plus saliva to analyse. The time from collecting the samples to preparation was minimized and the time from collecting the samples to they were frozen and stored did not exceed 60 min. All saliva samples as was all the blood samples, were handled in the same way, to avoid preanalytical errors.

### Proximity extension assay (PEA)

Briefly, 1 µL plasma from every aliquot was used in the OLINK Target 96 Inflammation panel analysis (Olink Bioscience, Uppsala, Sweden). The procedure was the same for the saliva samples. The samples were analysed according to the manufacturer’s instructions, applying a proximity extended assay (PEA) technology, where 92 inflammatory-related proteins were measured [19, 20]. The plasma was mixed with 3 µL of incubation mix, which contained two probes (antibodies labelled with unique corresponding DNA oligonucleotides). The mixture was incubated at 8 °C overnight. Following this, 96 µL of extension mix, containing the PEA enzyme and PCR reagents, was added. The samples were then incubated for 5 min at room temperature before being transferred to a thermal cycler for 17 cycles of DNA amplification. A 96.96 Dynamic Array IFC (Fluidigm, South San Francisco, CA, USA) was prepared and primed according to the manufacturer’s instructions. In a separate plate, 2.8 µL of the sample mixture was combined with 7.2 µL of detection mix, from which 5 µL was loaded into the right side of the primed 96.96 Dynamic Array IFC. Unique primer pairs for each cytokine were loaded into the left side of the 96.96 Dynamic Array IFC, and the protein expression program was run on the Fluidigm Biomark reader according to the Proseek instructions. Each Proseek kit measured 92 inflammatory CCGF biomarkers in total (Supplementary Table 6). The quantification of these protein concentrations is expressed logarithmically as normalized protein expression (NPX) [21]. The NPX value is an arbitrary unit on a Log2 scale meaning a difference in one unit of NPX corresponds to a doubling of the concentration.

### Statistics

All the output data from the OLINK set-up was utilized, analysing both saliva and plasma. 71 proteins in plasma, 63 proteins in saliva, and 58 proteins in both saliva and plasma out of 92 analysed showed >80% samples with NPX values above the limit of detection (LOD). LOD was defined as three standard deviations (SD) above the average of the negative controls. Spearman´s rank correlation coefficient was calculated using Statistica (StatSoft Version 14, Tulsa, OK, USA) to examine the correlation of expression of various cytokines (Table 4.) as NPX in saliva vs. plasma, (r_s_ = ± 1) [22]. For some descriptive parts of the data, Jamovi (ver. 2.3.28) was used.

When translating the numeric value into descriptive words, authors have chosen different words. For example, the r_s_ coefficient ± 0.2 or 0.3 has been described as weak, poor, moderate, or fair [23, 24]; in this study, we have chosen the word weak.

### Ethics

The Swedish Ethical Review Authority revised and approved the study protocol (Reference number: 2015/378. Date: 02/12/2015).

Prior to any study procedure, we obtained informed consent from all patients. Good Clinical Practice (GCP), General Data Protection Regulation (GDPR;2016/679), and the Helsinki Declaration were followed throughout the study.

## Results

One hundred and sixty-five patients, between 18 and 44 years old and weighing between 50 and 111 kg were included in this study (Table 2).

**Table 2:** Basic characteristics of the population (*N* = 165)

		Valid *N*	Mean	Median	Lower Quartile	Upper Quartile
Sex		165	112 females 53 males			
Age	year	165	29.1	29	23	35
Weight	kg	165	74.4	72.9	64.9	82.1
BMI	kg/m^2^	165	25.3	24.3	22.3	27.7
Hb	g/L	165	137	134	129	145
EVF		165	0.41	0.41	0.39	0.43
WBC	x10^9^/L	165	5.92	5.5	4.6	6.7
Plt	x10^9^/L	165	244	239	207	273
Alb	g/L	165	41.7	42	40	44
Crea	µmol/L	165	68.1	67	59	76

*EVF* Erytrocyte volume fraction, *WBC* White blood cell count, *Plt* Platelet count, *Alb* Albumin, Crea Creatinine

**Fig. 3:**
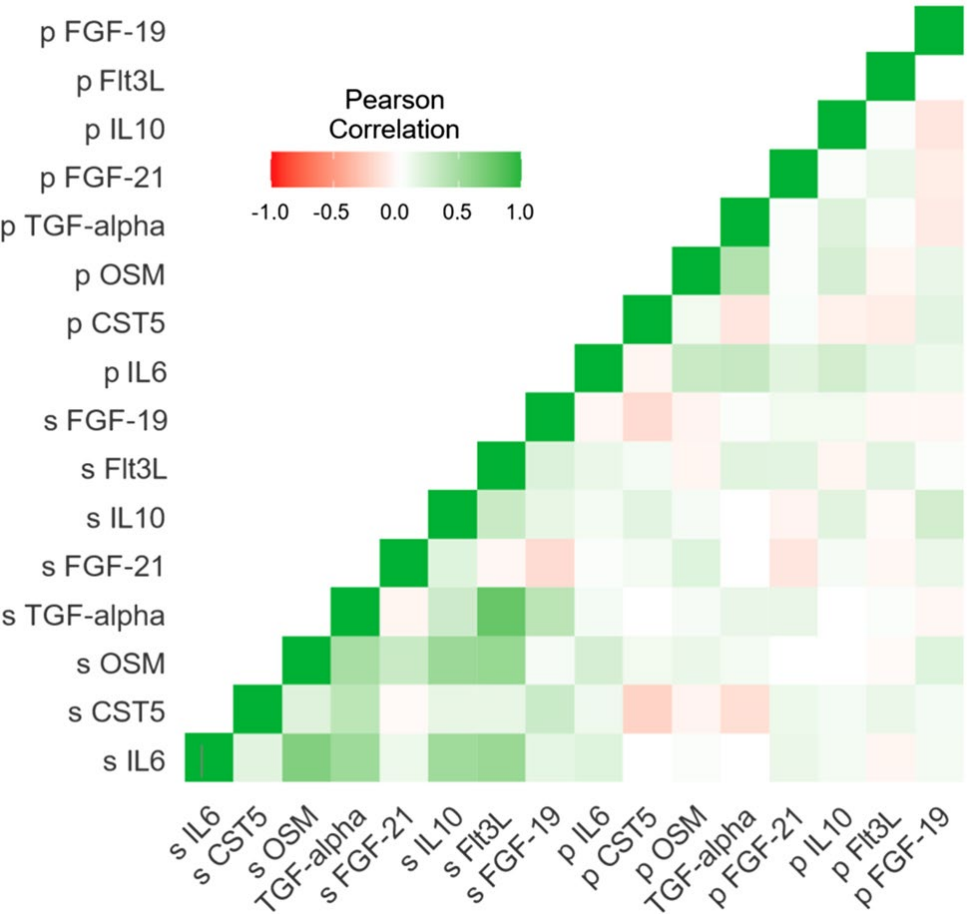
Heat map. Pearson correlation among 8 inflammatory biomarkers in saliva (s) vs. plasma (p) (Eriksson et al., submitted 2025).

**Fig. 4:**
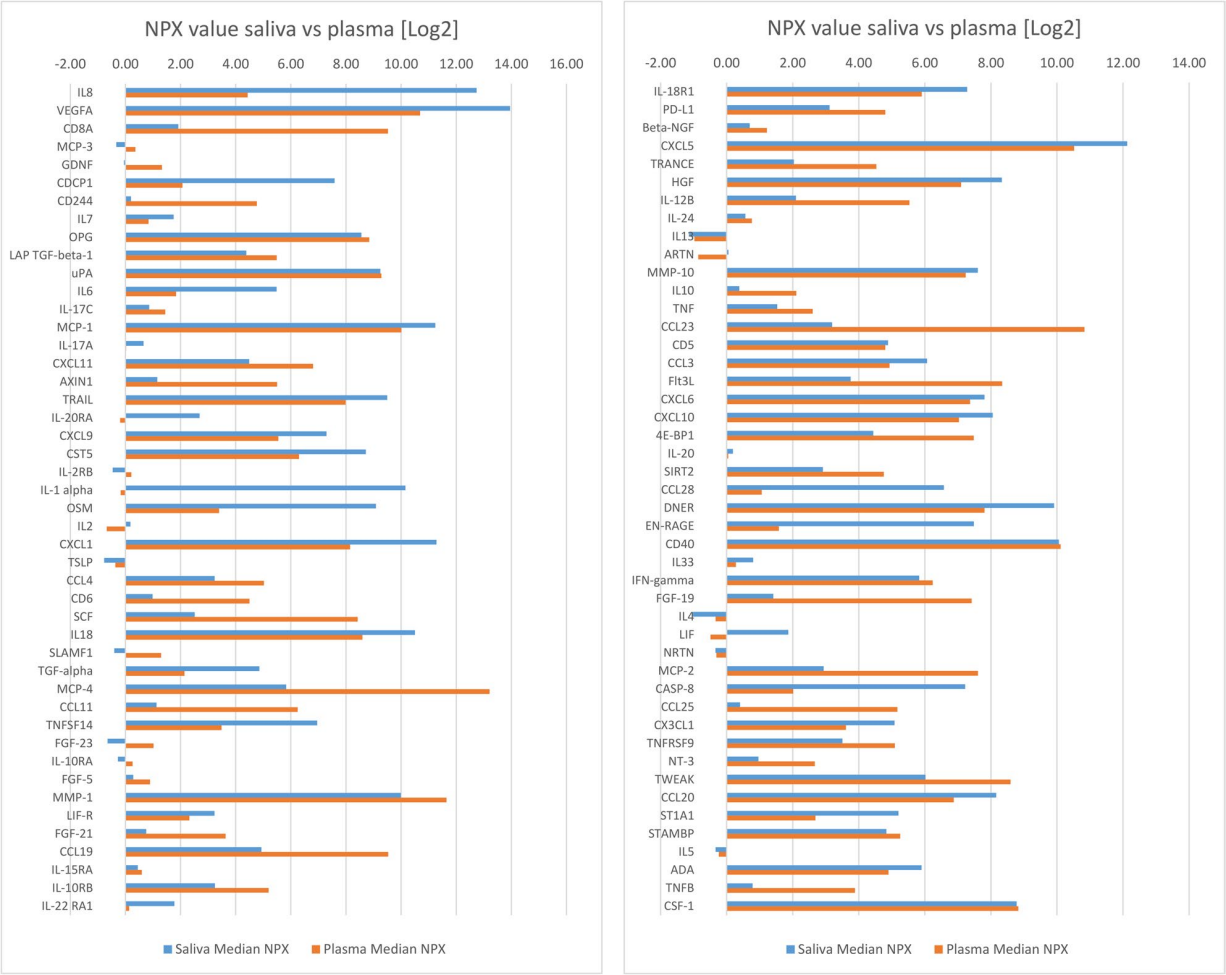
Relative expression of 92 inflammatory biomarkers. Proteins in saliva vs plasma samples collected simultaneously from study participants. The differences between saliva and plasma are noticeably large in some cases, considering that they are presented on a log2 scale

IL-6 and CST5 were significantly correlated to the same protein in saliva (Table 3.) (Fig. 3). Out of eight proteins in plasma, significantly altered by the surgical trauma of third molar surgery, only two correlated to the expression of the same protein in saliva (Eriksson et al. submitted 2025) (seen in Table 3.). Thirty-six correlated protein pairs between saliva and plasma were found when testing the eight saliva proteins (IL6, CST5, OSM, TGF-alpha, FGF-21, IL10, Flt3L, and FGF-19) against 92 different proteins in plasma. In only one pair the correlation was r_s_ ≥ ± 0.25, and in only six pairs the correlation was r_s_ ≥ ± 0.20. The p-value in all these cases was < 0.05. (Table 4.). When comparing median NPX-value for each protein in saliva vs. plasma (Fig. 4), four proteins uPA, CD40, NRTN, and CSF-1, were found to have a less than 5% difference in relative concentration between saliva and plasma (Table 5). When these four were tested for correlations using the Spearman rank scale, only weak correlations, r_s_=0.151 or less, were found (Table 5.). Some of the 92 CCGF were more strongly expressed in saliva than in plasma: IL-8, VEGFA, CDCP1, IL-6, IL-1 alpha, OSM, TNFSF14, CCL28, EN-RAGE, and CASP-8 (Fig. 4). Other CCGF were more strongly expressed in plasma: CD8A, CD244, AXIN1, SCF, MCP-4, FGF-21, CCL19, IL12B, CCL23, and FGF19 (Fig.4). A group of CCGF were weakly expressed in both saliva and plasma with NPX ≤ ± 1.0 (MCP-3, IL-17 A, IL-2RB, IL-2, TSLP, IL-10RA, FGF-5, IL-15RA, ARTN, IL-20, IL-33, and IL-5) (Fig.4).

**Table 3:** Comparison of eight biomarkers in plasma related surgical trauma and their expression in saliva

Saliva cytokine	Plasma cytokine	Uniprot	Spearman(*r*_s_)	*p*-value	s/ns
IL6	IL6	P05231	0.153554	0.048937	s
CST5	CST5	P28325	−0.254204	0.000985	s
OSM	OSM	P13725	0.076021	0.331802	ns
TGF-alpha	TGF-alpha	P01135	0.104004	0.183712	ns
FGF-21	FGF-21	Q9NSA1	0.079302	0.311299	ns
IL10	IL10	P22301	0.078783	0.315976	ns
Flt3L	Flt3L	P49771	0.105294	0.178308	ns
FGF-19	FGF-19	O95750	0.023853	0.761040	ns

In an earlier study (Eriksson et al., submitted 2025) we found eight cytokines in plasma that significantly increased or decreased pre- versus postoperatively due to third molar surgical trauma; these were tested for correlations, against the same cytokines in saliva (s/ns = significant/not significant)

**Table 4:** Inflammatory biomarkers with significant correlation between expression in saliva vs. plasma

Saliva cytokine	Uniprot-ID	Plasma cytokine	Spearman(*r*_s_)	*p*-value	s/ns
IL6	P05231	IL6	0.153554	0.048937	S
CST5	P28325	IL-17 A	0.153931	0.048378	S
CST5	P28325	CXCL11	0.171429	0.027692	S
CST5	P28325	CXCL9	0.155734	0.045778	S
CST5	P28325	CST5	−0.254204	0.000985	S
OSM	P13725	IL6	0.166711	0.032341	S
FGF-21	Q9NSA1	GDNF	−0.186047	0.016731	S
FGF-21	Q9NSA1	CXCL9	0.163984	0.035321	S
IL10	P22301	AXIN1	0.154051	0.048201	S
FGF-19	O95750	IL-17 A	−0.156349	0.044918	S
FGF-19	O95750	TRAIL	0.198042	0.010777	S
FGF-19	O95750	CST5	0.190500	0.014250	S
CST5	P28325	CCL11	0.186838	0.016264	S
FGF-21	Q9NSA1	OSM	−0.160254	0.039767	S
Flt3L	P49771	IL18	0.190564	0.014216	S
FGF-19	O95750	IL18	0.170855	0.028225	S
FGF-19	O95750	IL-15RA	0.191099	0.013942	S
FGF-19	O95750	IL-18R1	0.193329	0.012846	S
IL6	P05231	IL-24	0.190666	0.014164	S
FGF-21	Q9NSA1	ARTN	−0.158114	0.042524	S
FGF-21	Q9NSA1	CXCL10	0.212342	0.006179	S
Flt3L	P49771	CD5	0.156090	0.045279	S
FGF-19	O95750	DNER	0.195825	0.011711	S
IL6	P05231	CCL20	0.201867	0.009318	S
CST5	P28325	MCP-2	0.185895	0.016822	S
CST5	P28325	CCL20	0.199685	0.010127	S
OSM	P13725	MCP-2	−0.171520	0.027608	S
OSM	P13725	TNFB	−0.184818	0.017478	S
TGF-alpha	P01135	CCL25	0.177012	0.022938	S
TGF-alpha	P01135	TNFRSF9	0.206492	0.007791	S
FGF-21	Q9NSA1	IFN-gamma	0.161259	0.038525	S
IL10	P22301	FGF-19	−0.207023	0.007630	S
IL10	P22301	TNFB	−0.212160	0.006224	S
Flt3L	P49771	TNFRSF9	0.159100	0.041235	S
FGF-19	O95750	TNFRSF9	0.164184	0.035094	S
FGF-19	O95750	ST1A1	0.163751	0.035585	S

Eight CCGF in plasma (see Table 3.) were found to have significant changes due to surgical trauma (Eriksson et al., submitted 2025), and were tested for correlations to 92 CCGF in saliva. Of the 92 CCGF, 36 pairs from 8 × 92 possible combinations were found to have a correlation between saliva and plasma. Valid *N* = 165. s/ns = significant/not significant

**Table 5:** Ninety-two inflammatory proteins (CCGF) expressed as median NPX (log2) value in saliva vs plasma. Every step by one unit on the NPX log2 scale means a doubling of the difference. Proteins showing the least difference in concentration (<±5%) between saliva and plasma are marked with (*)

Protein	Saliva Median NPX Log2 scale	Plasma Median NPX Log2 scale	Least difference in concentration (< ± 5%) saliva vs. plasma (*)
IL8	12.74	4.43	
VEGFA	13.95	10.69	
CD8A	1.92	9.52	
MCP-3	−0.33	0.36	
GDNF	−0.05	1.32	
CDCP1	7.58	2.07	
CD244	0.20	4.76	
IL7	1.75	0.83	
OPG	8.56	8.85	
LAP TGF-beta-1	4.38	5.49	
uPA	9.25	9.28	*
IL6	5.48	1.84	
IL-17 C	0.86	1.44	
MCP-1	11.24	10.01	
IL-17 A	0.66	0.03	
CXCL11	4.49	6.81	
AXIN1	1.16	5.50	
TRAIL	9.50	7.98	
IL-20RA	2.69	−0.19	
CXCL9	7.30	5.54	
CST5	8.72	6.30	
IL-2RB	−0.46	0.21	
IL-1 alpha	10.16	−0.17	
OSM	9.09	3.40	
IL2	0.18	−0.68	
CXCL1	11.29	8.15	
TSLP	−0.77	−0.36	
CCL4	3.23	5.03	
CD6	0.98	4.50	
SCF	2.51	8.42	
IL18	10.51	8.60	
SLAMF1	−0.41	1.30	
TGF-alpha	4.86	2.14	
MCP-4	5.83	13.21	
CCL11	1.13	6.25	
TNFSF14	6.96	3.49	
FGF-23	−0.65	1.02	
IL-10RA	−0.27	0.26	
FGF-5	0.28	0.89	
MMP-1	9.99	11.65	
LIF-R	3.23	2.32	
FGF-21	0.75	3.63	
CCL19	4.93	9.53	
IL-15RA	0.45	0.60	
IL-10RB	3.25	5.19	
IL-22 RA1	1.78	0.14	
IL-18R1	7.28	5.91	
PD-L1	3.12	4.80	
Beta-NGF	0.70	1.22	
CXCL5	12.13	10.52	
TRANCE	2.04	4.53	
HGF	8.33	7.10	
IL-12B	2.10	5.53	
IL-24	0.57	0.76	
IL13	−1.14	−0.98	
ARTN	0.05	−0.86	
MMP-10	7.61	7.23	
IL10	0.38	2.11	
TNF	1.53	2.60	
CCL23	3.19	10.83	
CD5	4.89	4.80	
CCL3	6.07	4.93	
Flt3L	3.75	8.34	
CXCL6	7.81	7.37	
CXCL10	8.05	7.04	
4E-BP1	4.44	7.48	
IL-20	0.19	0.05	
SIRT2	2.91	4.76	
CCL28	6.58	1.06	
DNER	9.91	7.81	
EN-RAGE	7.48	1.58	
CD40	10.06	10.11	*
IL33	0.80	0.28	
IFN-gamma	5.83	6.24	
FGF-19	1.41	7.42	
IL4	−1.03	−0.34	
LIF	1.87	−0.49	
NRTN	−0.34	−0.31	*
MCP-2	2.93	7.61	
CASP-8	7.22	2.01	
CCL25	0.41	5.17	
CX3CL1	5.08	3.61	
TNFRSF9	3.51	5.09	
NT-3	0.96	2.67	
TWEAK	6.02	8.60	
CCL20	8.16	6.88	
ST1A1	5.20	2.69	
STAMBP	4.84	5.25	
IL5	−0.33	−0.24	
ADA	5.90	4.90	
TNFB	0.78	3.89	
CSF-1	8.78	8.83	*

## Discussion

This extended study supports previous results, suggesting that the correlation between saliva and plasma regarding expression of inflammatory biomarkers is rather weak. The reasons why the concentrations of the same protein might differ between saliva and plasma are multiple. For example, saliva and plasma are separate compartments with different purposes. The expression of proteins in saliva may suffer from dilution effects due to stimulated and non-stimulated secretion and also influenced by the microenvironment in the mouth with its presence of microbes is different from plasma, which is supposed to be sterile. The method of analysing large numbers of protein from small sample volumes are highly specific and expressed in a relative strength of expression (NPX) which is accurate but lack the intuitive understanding of an absolute concentration. With that said comparisons between different matrix such as saliva and plasma are as correct as comparisons within one matrix [19, 20, 25]. This study found only four proteins with small differences (< ± 5%) in NPX-value between saliva and plasma; urokinase-type plasminogen activator (uPA), cluster of differentiation 40 (CD40), neurturin (NRTN), and colony-stimulating factor 1(CSF-1).

uPA is related to proteolysis in cancer invasion and metastasis [26]. CD40 is involved in regulation of B-cell responses and can also be expressed on dendritic cells, macrophages, fibroblasts and endothelial cells [27]. CD40 is involved in cell mediated immunity [28]. The expression of CD40 in the oral epithelium is restricted to keratinocytes and are inter- and intra-individually variated [28]. Neurturin is found to activate and sensitize bone afferent neurons inducing pain associated with bone pathology [29]. There are indications that CD40 as well as CSF-1 is elevated in painful diabetic neuropathy [30].

### Prominent findings in saliva

This study showed that IL-6 in saliva and plasma and CST5 in saliva and plasma are both statistically significantly correlated, even though the correlations are weak. The study also showed 36 additional heterogeneous cytokine pair combinations to be significantly correlated between saliva and plasma, although again, the correlations remained weak. Nevertheless, some proteins were found to be much more strongly expressed in saliva, which may be the result of unknown patterns in the immune system. The most prominent proteins in saliva that we found were involved in a range of activities, such as angiogenesis, immune response and inflammation signalling, as well as apoptosis. IL-8 is a pro-inflammatory chemokine that primarily attracts and activates neutrophils [31]. The vascular endothelial growth factor A (VEGFA) promotes angiogenesis [32]. VEGFA regulates the formation of new blood vessels from existing ones [33]. IL-6 levels increase early (within an hour or faster) as a response to tissue damage caused by trauma or surgery, for instance [34]. The level of IL-6 is associated with the severity of the tissue damage [35]. Further, IL-6 has been shown to be essential in wound healing by modulating immune system activity [36]. IL-1 alpha is part of a group of proteins called alarmins. The group includes high-mobility group box 1 protein (HMGB1), IL-1 alpha, IL-33, and Ca^2+^ binding S100 [37]. The IL-1 family contains 11 members. In the development of inflammatory diseases and cancer, IL1-alpha plays a key role among the other alarmins [37]. IL-1 alpha is a dual function cytokine with both intracellular and extracellular functions. It is expressed in epithelial cells in the gastrointestinal tract, skin, liver, and kidney [37]. Release of IL-1 alpha leads to chemokine secretion with neutrophil infiltration [37]. Oncostatin M (OSM) is a part of the IL-6 family in which all members are important for cell signaling and cell communication in the immune system [38]. OSM is known to have protective effects on myelination, which is important for signal transduction along axons and for axonal protection [38]. TNF superfamily 14 (TNFSF14) is also known as LIGHT [39]. TNFSF14 is a transmembrane glycoprotein that plays a central role in the acute innate immune response [39]. TNFSF14 is involved in atherosclerosis and vascular inflammation [39]. CCL28 is a member of the chemokine family and activates immune cells throughout the body [40]. Due to its expression in epithelium and mucosal secretion, such as through milk and saliva, CCL28 may provide innate immune defence against a variety of bacterial pathogenes [40]. The highest levels of CCL28 expression can be found in salivary glands [40] and are increased in response to inflammatory events, such as rheumatoid [40]. RAGE is an immunoglobulin expressed in multiple tissues such as endothelium, vascular smooth muscle cells, and monocyte derived macrophages [41]. The activation of inflammatory cascades is a result of the binding of RAGE by EN-RAGE [41]. EN-RAGE facilitates inflammatory monocyte activation. EN-RAGE is a member of the S100 protein family [41]. Previous studies have observed increased levels of EN-RAGE in chronic inflammatory disorders. CASP-8 plays a role in initiating extrinsic apoptosis [42]. Apoptosis, programmed cell death [43], can be initiated by treatment with TNF, which activates CASP-8 [42].

### Prominent findings in plasma

Some inflammatory biomarkers were more prominently expressed in plasma than saliva: CD8A, CD244, AXIN1, SCF, MCP-4, FGF-21, CCL19, IL-12B, CCL23, and FGF19 CD8/CD8 T-cell response are associated with the immune system´s antiviral response [44]. It is known that CD244 signalling correlates with certain virus persistence in humans, namely hepatitis B, hepatitis C, and tuberculosis (TB) [45]. In active human TB, CD244 signalling regulates repression of IFN-gamma and IFN alpha [45]. AXIN1 has been thought to be a tumour suppressor protein, although its function remains largely undefined [46]. SKP1-CUL1-F-box protein (SCF) has mostly been associated with cell proliferation, survival, and the connection to cancer. Disorders related to sleep, mood, metabolism, and intellect, need to be studied to better understand the function of SCF [47]. Monocyte chemoattractant protein (MCP)−4 is a pro-inflammatory protein overexpressed in many malignant tumours and may be important in the progression of tumours and metastasis [48]. Together with FGF-19 and FGF-23, fibroblast growth factor (FGF)−21 forms a subfamily [49]. FGF-21 is related to typ 2 diabetes, metabolic syndrome, coronary heart disease, obesity, and chronic kidney disease [50]. FGF-19 is reduced in diabetes and obesity and is inversely correlated to BMI shown in patients going through bariatric surgery [51, 52]. Chemokine receptor ligand (CCL)−19 promotes both breast- and cervical cancer progression. There are also indications that CCL19 suppresses lung cancer, colorectal cancer, ovarian cancer, and gastric cancer [53]. As a pro inflammatory cytokine, IL-12 is crucial for the antiviral immune response [54]. Both amount and variability of cytokine synthesis are genetically regulated by single nucleotide polymorphisms (SNPs) in IL-12 A and IL-12B; these also influence susceptibility to infectious diseases, disease severity, as well as the response to antiviral treatment [54]. Chemokine receptor ligand (CCL)−23 is associated with the outcome of stroke and acquired brain damage. Chemokines in the CC subgroup can attract basophils, monocytes, eosinophils, T lymphocytes, dendritic cells, and natural killer (NK) cells. CCL23 inhibits both production and release of monocytes and polymorphonuclear cells (PMNs) in bone marrow [55].

### Biomarkers in saliva

Williams et al. compared two different samplings techniques for saliva and the levels of 27 cytokines in saliva and plasma, in 50 participants [1]. Williams et al. found that the correlations between saliva and plasma cytokine levels were not robust enough to allow substitution. As a result, they recommended that caution should be used in substituting saliva for plasma, and to consider that relationships can vary depending on the specific biomarker [1]. A study from 2021, of 43 adolescent competitive swimmers, which measured cytokines in saliva and plasma before and after exercise, states similar conclusions [2]. In this study, IL-6 levels in saliva and plasma differed significantly, with higher concentrations observed in saliva [2]. In another study, forty-eight different CCGF were analysed in saliva, plasma, and urine, in twenty healthy volunteers [4]. Thirty-seven of 48 CCGFs were found in plasma, 41 in saliva, and 34 in urine; this study reported the absolute concentration of each CCGF, but no correlation analysis was reported [4]. In a recent study of inflammatory biomarkers in children with juvenile idiopathic arthritis (JIA), saliva and serum were compared using the inflammatory panel from OLINK [56]. Cetrelli et al. concluded that the difference in biomarker patterns between saliva and serum is too large to justify using saliva as a substitute for plasma when assessing the degree of inflammation [56]. By analysing serum instead of plasma Cetrelli et al. offer a different perspective from other articles on the subject [56]. Serum lacks the coagulation factors still present in plasma, which makes the results not entirely comparable [18]. In a small study on children from 2021, IL-6, IL-10 and TNF-alpha were compared in saliva and plasma, which led to the conclusion that salivary measurement of cytokines is not a sufficient substitute for plasma, due to a weak correlation between these two fluids [2]. When investigating the inflammatory profile in patients with neuropathic pain, the most significant biomarkers for separating the groups was found in saliva as well in plasma and cerebrospinal fluid [57]. Jonsson et al. conclude that YKL-40 and MIP-1-alpha in saliva indicate a potential for further investigation [57]. Various authors have studied the possibility of using saliva as screening material for specific diseases, such as oral cancer [9], periodontitis [58], Alzheimer´s disease [59], amyotrophic lateral sclerosis (ALS) [60], cardiovascular disease [61], recurrent respiratory tract infections (rRTIs) in young children [62], and pregnancy outcome [63]. Majster et al. show increased IL-6 and MMP-10 levels in saliva in active inflammatory bowel disease [64]. It is well recognised that saliva and plasma differ in the expression of certain biomarkers. The strength of this study lies in its larger cohort and broader range of biomarkers, thereby confirming the conclusions of other authors [2, 18, 56].

### Strength and limitations

The number of patients enrolled in this study is a strength, as is the fact that saliva and plasma were collected from the same individuals on the same occasion. The consequent procedure when collecting and preparing the biological samples is thus a strength. Another strength is the broad number of proteins analysed in comparison with similar, smaller studies [3]. All samples of saliva and plasma were handled consequently and according to a written routine to minimize the risk of any preanalytical error. There are some limitations with our study, however. First, this is a single site study. Second, there is an imbalance in sex representation, since 2/3 of the participants are women. Third, the study does not include children or elderly people, thus the age span is limited. Fourth, the analysis method (OLINK) is very sensitive and precise; for this reason, the results are reported as relative concentrations (NPX) rather than absolute concentrations (pg/ml). Fifth, no protease inhibitor was added to the saliva during sample collection, which means that some degree of proteolysis may have occurred in the samples. The exact consequence of not using protease inhibitors cannot be quantifies by our method. Sixth, although several biomarkers were studied, there could be numerous proteins of significant relevance to the topic that are not included in the selected panel. Seventh, the saliva and plasma samples were collected between 8 am and 2 pm, the intra individual range was only a few minutes. The inter individual differences may be caused by circadian variation, but we believe that the relatively large number of participants and the randomization procedures eliminate most of the risk of unrepresentative results. Lastly, since the study is cross-sectional, no conclusions regarding causality can be drawn and no conclusions can be drawn regarding physiological compliance between saliva and plasma.

### Positive and negative control for unspecific binding awareness in the proximity extended assay (PEA) technology

Olink assays are developed with an emphasis on specificity and reducing non-specific binding. The cornerstone of the assay specificity is the dual antibody recognition and high-fidelity DNA-coupled measurement in the PEA protocol.

For each protein target, two oligonucleotide-coupled antibodies (PEA probes) must bind in close enough proximity to enable the oligos to hybridize and form a unique DNA template for detection. This overcomes the problems normally associated with multiplexed immunoassays,

The internal controls are spiked into every sample and are designed to monitor the three steps of the Olink protocol (immuno, extension and detection controls): *Incubation control* (immuno control) is non-human antigen that measured with PEA. Immuno control monitors potential technical variation in all three steps of the PEA reaction. *The Extension Control* is composed of an antibody coupled to a unique pair of DNA-tags. *The Detection Control* is a complete double stranded DNA amplicon which does not require any proximity binding or extension step to generate a signal. This control monitors the amplification/detection step.

Additionally, each sample plate contains a designated row of external controls. Sample control: *Negative Control* is also included in triplicate on each plate and consists of buffer run as a normal sample. These are used to monitor any background noise generated when DNA-tags come in close proximity without prior binding to the appropriate protein. The negative controls set the background levels for each protein assay and are used to calculate the limit of detection (LOD). *Inter-plate Control (IPC)* is included in triplicate on each plate, and these are run as normal samples. The median of the IPC triplicates is used to normalize each assay, to compensate for potential variation between runs and plates [19, 20, 25].

## Conclusions

We found major differences in the levels of inflammatory biomarkers in saliva vs. plasma measured by the OLINK method. The main differences were that out of the ten most prominent proteins in saliva, six are especially interesting for further investigation. IL-6 and IL-8 are both pro-inflammatory cytokines. IL-6 and OSM belong to the same family; IL-1 alpha is part of another cytokine family and is anti-inflammatory. IL-6 is induced by IL-1 alpha, among several others. CST5 is a proteases inhibitor first found in saliva, and which modulates the whole inflammatory cascade by influencing the cytokine production. CCL28 is a chemokine with the highest level of expression in salivary glands. We also found inflammatory biomarkers in saliva that are weakly correlated with biomarkers in plasma: IL-6 and CST5. To summarize, saliva and plasma are not interchangeable when monitoring inflammatory biomarkers. This information may be important when planning for future inflammation studies.

## Supplementary Information

Supplementary Material 1.

## Data Availability

The datasets used and/or analysed during the current study are available from the corresponding author on reasonable request.

## Abbreviations

Alb: Albumin
AXIN: Axin
BMI: Body mass index
CASP: Caspase
CCGF: Cytokines, chemokines, and growth factors
CCL: Chemokine receptor ligand
CD: T-cell surface glycoprotein
CDCP: CUB domain-containing protein
OSM: Oncostatin M
CLCF: Cardiotrophin-like cytokine factor
CNTF: Ciliary neurotrophic factor
Crea: Creatinine
CSF: Macrophage colony-stimulating factor
CST5: Salivary cystatin D
CT: Creatine transporter
EDTA: Ethylenediaminetetraacetic acid
EN-RAGE: Protein S100-A12
EudraCT: European Union Drug Regulating Authorities Clinical Trials Database
EVF: Erytrocyte volume fraction
FasL: Fas Ligand
FGF: Fibroblast growth factor
Flt3L: FMS-related tyrosine kinase 3 ligand
GCP: Good clinical practice
GDPR: General data protection regulation
IFN: Interferon
IL: Interleukin
IV: Intravenous
kD: kilo Dalton
LIF: Leukemia inhibitory factor
LOD: Limit of detection
MCP: Monocyte chemoattractant protein
N: Number
NPX: Normalized protein expression
NRTN: Neurturin
PCR: Polymerase chain reaction
PDGF: Platelet derived growth factor
PEA: Proximity extension assay
Plt: Platelet count
RAGE: Receptor of advanced glycation end products
r_s_: Spearman rank coefficient
s/ns: Significant/not significant
SCF: Stem cell factor
SD: Standard deviation
TFG: Transforming growth factor
TGF: Tumour growth factor
TNF: Tumour necrosis factor
TNFSF: Tumour necrosis factor ligand superfamily member
uPA: Urokinase-type plasminogen activator
VEGFA: Vascular endothelial growth factor A
WBC: White blood cell count
