# Supplementary material for: Methodological comparison between salivary and plasma inflammatory biomarkers in third molar surgery patients
Source: BMC Oral Health. 2025 Nov 22;25:1902. doi: 10.1186/s12903-025-07368-2 (PMC12703913; doi:10.1186/s12903-025-07368-2)
Supplement: Supplementary file 1 — Supplementary Material 1. [file 12903_2025_7368_MOESM1_ESM.docx]

**Supplementary Table 6.** List of cytokines, chemokines, and growth

factors analyzed, using the Proseek Multiplex Inflammation

kit version 3024 (Olink Bioscience, Uppsala, Sweden).

Analyzed in saliva and plasma.

| Assay/Protein | UniProtID |
| --- | --- |
| Adenosine Deaminase (ADA) | P00813 |
| Artemin (ARTN) | Q5T4W7 |
| Axin-1 (AXIN1) | O15169 |
| Beta-nerve growth factor (Beta-NGF) | P01138 |
| Caspase-8 (CASP-8 ) | Q14790 |
| C-C motif chemokine 19 (CCL19) | Q99731 |
| C-C motif chemokine 20 (CCL20) | P78556 |
| C-C motif chemokine 23 (CCL23) | P55773 |
| C-C motif chemokine 25 (CCL25) | O15444 |
| C-C motif chemokine 28 (CCL28) | Q9NRJ3 |
| C-C motif chemokine 3 (CCL3) | P10147 |
| C-C motif chemokine 4 (CCL4 ) | P13236 |
| CD40L receptor (CD40) | P25942 |
| CUB domain-containing protein 1 (CDCP1) | Q9H5V8 |
| C-X-C motif chemokine 1 (CXCL1) | P09341 |
| C-X-C motif chemokine 10 (CXCL10 ) | P02778 |
| C-X-C motif chemokine 11 (CXCL11) | O14625 |
| C-X-C motif chemokine 5 (CXCL5 ) | P42830 |
| C-X-C motif chemokine 6 (CXCL6) | P80162 |
| C-X-C motif chemokine 9 (CXCL9 ) | Q07325 |
| Cystatin D (CST5) | P28325 |
| Delta and Notch-like epidermal growth factor-related receptor (DNER) | Q8NFT8 |
| Eotaxin (CCL11) | P51671 |
| Eukaryotic translation initiation factor 4E-binding protein 1 (4E-BP1) | Q13541 |
| Fibroblast growth factor 19 (FGF-19) | O95750 |
| Fibroblast growth factor 21 (FGF-21) | Q9NSA1 |
| Fibroblast growth factor 23 (FGF-23) | Q9GZV9 |
| Fibroblast growth factor 5 (FGF-5) | P12034 |
| Fms-related tyrosine kinase 3 ligand (Flt3L) | P49771 |
| Fractalkine (CX3CL1 ) | P78423 |
| Glial cell line-derived neurotrophic factor (GDNF) | P39905 |
| Hepatocyte growth factor (HGF) | P14210 |
| Interferon gamma (IFN-gamma) | P01579 |
| Interleukin-1 alpha (IL-1 alpha) | P01583 |
| Interleukin-10 (IL10) | P22301 |
| Interleukin-10 receptor subunit alpha (IL-10RA) | Q13651 |
| Interleukin-10 receptor subunit beta (IL-10RB) | Q08334 |
| Interleukin-12 subunit beta (IL-12B) | P29460 |
| Interleukin-13 (IL-13) | P35225 |
| Interleukin-15 receptor subunit alpha (IL-15RA) | Q13261 |
| Interleukin-17A (IL-17A) | Q16552 |
| Interleukin-17C (IL-17C) | Q9P0M4 |
| Interleukin-18 (IL-18) | Q14116 |
| Interleukin-18 receptor 1 (IL-18R1) | Q13478 |
| Interleukin-2 (IL-2) | P60568 |
| Interleukin-2 receptor subunit beta (IL-2RB) | P14784 |
| Interleukin-20 (IL-20) | Q9NYY1 |
| Interleukin-20 receptor subunit alpha (IL-20RA) | Q9UHF4 |
| Interleukin-22 receptor subunit alpha-1 (IL-22 RA1) | Q8N6P7 |
| Interleukin-24 (IL-24) | Q13007 |
| Interleukin-33 (IL-33) | O95760 |
| Interleukin-4 (IL-4) | P05112 |
| Interleukin-5 (IL-5) | P05113 |
| Interleukin-6 (IL-6) | P05231 |
| Interleukin-7 (IL-7) | P13232 |
| Interleukin-8 (IL-8) | P10145 |
| Latency-associated peptide transforming growth factor beta-1 (LAP TGF-beta-1) | P01137 |
| Leukemia inhibitory factor (LIF) | P15018 |
| Leukemia inhibitory factor receptor (LIF-R) | P42702 |
| Macrophage colony-stimulating factor 1 (CSF-1) | P09603 |
| Matrix metalloproteinase-1 (MMP-1) | P03956 |
| Matrix metalloproteinase-10 (MMP-10) | P09238 |
| Monocyte chemotactic protein 1 (MCP-1) | P13500 |
| Monocyte chemotactic protein 2 (MCP-2) | P80075 |
| Monocyte chemotactic protein 3 (MCP-3) | P80098 |
| Monocyte chemotactic protein 4 (MCP-4) | Q99616 |
| Natural killer cell receptor 2B4 (CD244) | Q9BZW8 |
| Neurotrophin-3 (NT-3) | P20783 |
| Neurturin (NRTN) | Q99748 |
| Oncostatin-M (OSM) | P13725 |
| Osteoprotegerin (OPG) | O00300 |
| Programmed cell death 1 ligand 1 (PD-L1) | Q9NZQ7 |
| Protein S100-A12 (EN-RAGE ) | P80511 |
| Signaling lymphocytic activation molecule (SLAMF1) | Q13291 |
| SIR2-like protein 2 (SIRT2) | Q8IXJ6 |
| STAM-binding protein (STAMPB) | O95630 |
| Stem cell factor (SCF) | P21583 |
| Sulfotransferase 1A1 (ST1A1) | P50225 |
| T cell surface glycoprotein CD6 isoform (CD6) | P30203 |
| T-cell surface glycoprotein CD5 (CD5) | P06127 |
| T-cell surface glycoprotein CD8 alpha chain (CD8A) | P01732 |
| Thymic stromal lymphopoietin (TSLP) | Q969D9 |
| TNF-beta (TNFB) | P01374 |
| TNF-related activation-induced cytokine (TRANCE) | O14788 |
| TNF-related apoptosis-inducing ligand (TRAIL) | P50591 |
| Transforming growth factor alpha (TGF-alpha) | P01135 |
| Tumor necrosis factor (Ligand) superfamily, member 12 (TWEAK) | O43508 |
| Tumor necrosis factor (TNF) | P01375 |
| Tumor necrosis factor ligand superfamily member 14 (TNFSF14 ) | O43557 |
| Tumor necrosis factor receptor superfamily member 9 (TNFRSF9) | Q07011 |
| Urokinase-type plasminogen activator (uPA) | P00749 |
| Vascular endothelial growth factor A (VEGF-A) | P15692 |
